# *N*-[2-(2,2-Di­methyl­propanamido)­pyrimidin-4-yl]-2,2-di­methyl­propanamide *n*-hexane 0.25-solvate hemihydrate

**DOI:** 10.1107/S160053681302713X

**Published:** 2013-10-05

**Authors:** Borys Ośmiałowski, Arto Valkonen, Lilianna Chęcińska

**Affiliations:** a Faculty of Technology and Chemical Engineering, University of Technology and Life Sciences, Seminaryjna 3, PL-85-326 Bydgoszcz, Poland; b Department of Chemistrycv5431, University of Jyväskylä, P.O. Box 35, FI-40014 Jyväskylä, Finland; c Structural Chemistry and Crystallography Group, University of Lodz, Pomorska 163/165, PL-90-236 Łódź, Poland

## Abstract

The asymmetric unit of the title compound, C_14_H_22_N_4_O_2_·0.25C_6_H_14_·0.5H_2_O, contains two independent mol­ecules of 2,4-bis­(pivaloyl­amino)­pyrimidine (*M*) with similar conformations, one water mol­ecule and one-half *n*-hexane solvent mol­ecule situated on an inversion center. In one independent *M* mol­ecule, one of the two *tert*-butyl groups is rotationally disordered between two orientations in a 3:2 ratio. The *n*-hexane solvent mol­ecule is disordered between two conformations in the same ratio. The water mol­ecule bridges two independent *M* mol­ecules *via* O—H⋯O, N—H⋯O and O—H⋯N hydrogen bonds into a 2*M*·H_2_O unit, and these units are further linked by N—H⋯N hydrogen bonds into chains running in the [010] direction. Weak C—H⋯O inter­actions are observed between the adjacent chains.

## Related literature

For the related structures of 2,4-bis­(acyl­oamino)­pyrimidines in the solid state and in solution, see: Ośmiałowski *et al.* (2012 ▶). For the related structures of 2,6-bis­(acyl­oamino)­pyridines, see: Ośmiałowski *et al.* (2010 ▶); Crane (2003 ▶).
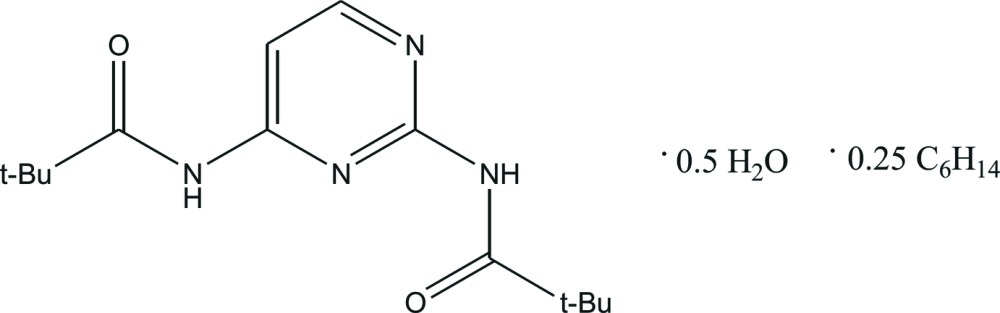


## Experimental

### 

#### Crystal data

2C_14_H_22_N_4_O_2_·0.5C_6_H_14_·H_2_O
*M* *_r_* = 617.81
Triclinic, 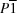

*a* = 10.6055 (5) Å
*b* = 12.2181 (6) Å
*c* = 14.9774 (7) Å
α = 88.060 (3)°
β = 73.093 (4)°
γ = 74.179 (3)°
*V* = 1784.36 (16) Å^3^
*Z* = 2
Mo *K*α radiation
μ = 0.08 mm^−1^
*T* = 123 K
0.30 × 0.05 × 0.04 mm

#### Data collection

Bruker–Nonius KappaCCD diffractometer with an APEXII detector
Absorption correction: multi-scan (*SADABS*; Sheldrick, 2004 ▶) *T*_min_ = 0.977, *T*_max_ = 0.997
21621 measured reflections
6422 independent reflections
3597 reflections with *I* > 2σ(*I*)
*R*_int_ = 0.100

#### Refinement

*R*[*F* ^2^ > 2σ(*F* ^2^)] = 0.079
*wR*(*F* ^2^) = 0.164
*S* = 1.04
6422 reflections
451 parameters
101 restraints
H atoms treated by a mixture of independent and constrained refinement
Δρ_max_ = 0.35 e Å^−3^
Δρ_min_ = −0.24 e Å^−3^

### 

Data collection: *COLLECT* (Bruker, 2008 ▶); cell refinement: *DENZO-SMN* (Otwinowski & Minor, 1997 ▶); data reduction: *DENZO-SMN*; program(s) used to solve structure: *SIR2004* (Burla *et al.*, 2005 ▶); program(s) used to refine structure: *SHELXL2013* (Sheldrick, 2008 ▶); molecular graphics: *PLATON* (Spek, 2009 ▶); software used to prepare material for publication: *SHELXL2013* and *publCIF* (Westrip, 2010 ▶).

## Supplementary Material

Crystal structure: contains datablock(s) I, global. DOI: 10.1107/S160053681302713X/cv5431sup1.cif

Structure factors: contains datablock(s) I. DOI: 10.1107/S160053681302713X/cv5431Isup2.hkl

Supplementary material file. DOI: 10.1107/S160053681302713X/cv5431Isup3.cml

Additional supplementary materials:  crystallographic information; 3D view; checkCIF report

## Figures and Tables

**Table 1:** Hydrogen-bond geometry (Å, °)

*D*—H⋯*A*	*D*—H	H⋯*A*	*D*⋯*A*	*D*—H⋯*A*
O1—H1*A*⋯O8*A*	0.84 (2)	2.08 (2)	2.910 (3)	167 (4)
O1—H1*A*⋯N1*A*	0.84 (2)	2.51 (4)	2.958 (4)	115 (3)
O1—H1*B*⋯O8	0.83 (2)	2.13 (2)	2.943 (3)	168 (4)
O1—H1*B*⋯N1	0.83 (2)	2.48 (4)	2.931 (4)	115 (3)
N7—H7⋯N3*A* ^i^	0.88 (2)	2.32 (2)	3.144 (4)	156 (3)
N13—H13⋯O1	0.87 (2)	2.02 (2)	2.864 (4)	162 (4)
N7*A*—H7*A*⋯N3^ii^	0.87 (2)	2.16 (2)	2.958 (4)	152 (3)
N13*A*—H13*A*⋯O1	0.89 (2)	2.02 (2)	2.882 (4)	164 (4)
C5—H5⋯O14^iii^	0.95	2.37	3.205 (5)	147

Symmetry codes: (i) 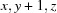; (ii) 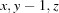; (iii) 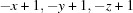.

**Fig. 1.**
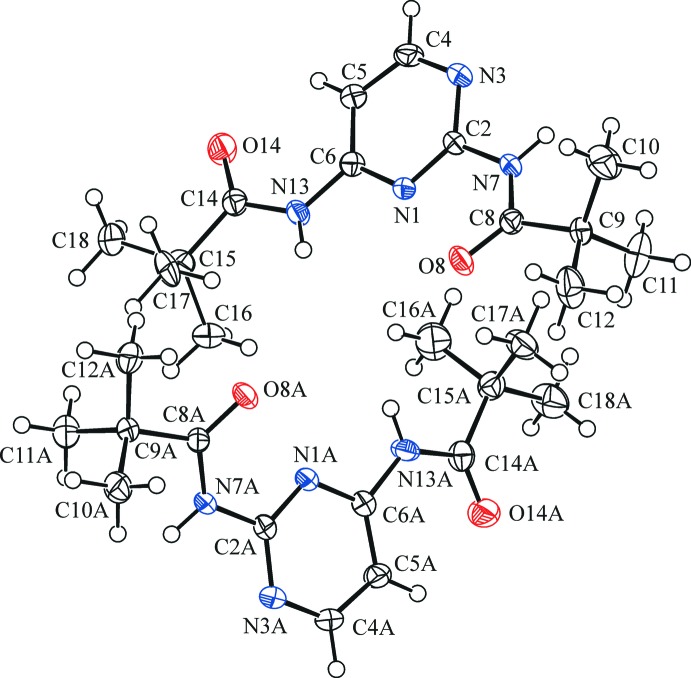
Two independent molecules M in (I), with atom labels and 50% probability displacement ellipsoids for non-H atoms. Only the major component of disordered t-butyl group is shown. Water and hexane solvent molecules are omitted for clarity.

**Fig. 2.**
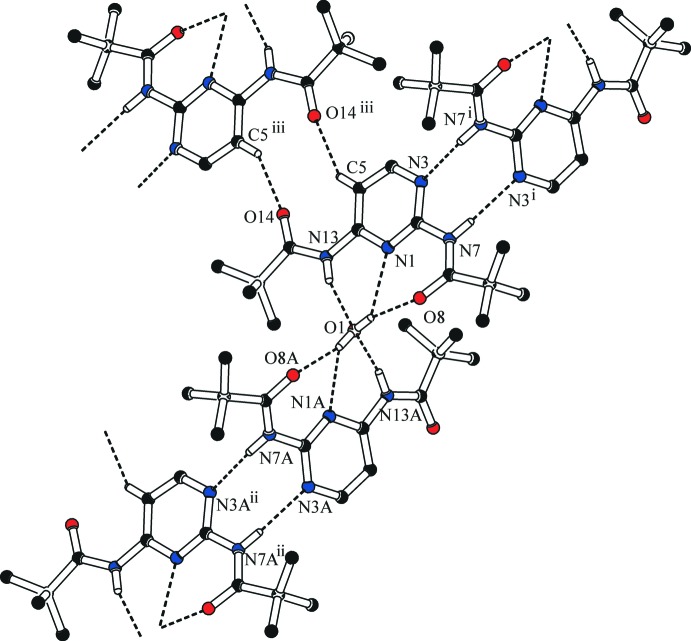
Part of the crystal structure of (I) showing the intermolecular hydrogen-bonds [symmetry codes: (i) x, 1 + y, z; (ii) x, y - 1, z; (iii) 1 - x, 1 - y,1 - z]. Hexane solvent molecule, the minor components of disordered t-butyl group and (C)—H atoms are omitted for clarity.

## supplementary crystallographic information

### 1. Comment

The 2,4-bis(pivaloylamino)pyrimidine is able to form various rotamers in solution (Ośmiałowski *et al.*, 2012). The high flexibility of this molecule with respect to the related pyridine derivatives is caused by the presence of two nitrogen atoms within the aromatic ring. This was confirmed by studies of association of such derivatives with various supramolecular counterparts (Ośmiałowski *et al.*, 2010; 2012). In the crystal structure of the related pyridine derivative (2,6-bis(pivaloylamino)pyridine) a weak N—H···O═C interaction was found, which further links the molecules into chain motif (Crane, 2003).

The 2,4-bis(pivaloylamino)pyrimidine was crystallized from *n*-hexane as hemisolvate monohydrate. In the asymmetric unit there are two independent molecules of the title compound (Figure 1). Water molecule interacts with these molecules *via* O—H···O, N—H···O and O—H···N intermolecular hydrogen bonds (Table 1) thereby joins them into hydrogen-bonded dimer (Figure 2). It is worth mentioning that water molecule incorporated between two subjected molecules forces those to adopt the *Z*,*E*,*Z*,*Z* conformation (Ośmiałowski *et al.*, 2012), in which the electron repulsion between heterocyclic nitrogen atoms (N1, N1A) and carbonyl oxygen atoms (O8, O8A) are observed.

Furthermore, each independent molecule forms an intermolecular N—H···N hydrogen bond (Table 1), thus producing infinite chain of dimers running parallel to the [010] direction. Two centrosymmetrically related chains interact each other *via* weak C—H···O interactions (Table 1).

The partially disordered *n*-hexane solvent molecule lies on the inversion centre. In the crystal lattice it is surrounded by the terminal *t*-butyl groups. This arrangement precludes any significant intermolecular interactions with other molecules in crystal.

### 2. Experimental

The title compound was synthesized according to the method of Ośmiałowski *et al.* (2012). Crystals suitable for X-ray measurements were obtained by crystallization from *n*-hexane.

### 3. Refinement

All non-hydrogen atoms were refined anisotropically. One *t*-butyl group (atoms C16, C17, C18) of molecule 1 as well as some atoms of hexane solvent molecule (C20 and all H-atoms) were refined as disordered over two sets of sites with occupancies fixed at 0.60:0.40. The C—C bond distances within the disordered *t*-butyl group as well as the C—C distances between disordered atoms within hexane moiety (pairs: C19—C20/C19—C20 and C21—C20/C21—C20A, respectively) were restrained to be approximately equal. Moreover, the *U_ij_* components of atoms in two *t*-butyl groups in molecule 1 were restrained to be similar. Additionally, the ADPs of the C16B atom were restrained to be approximately isotropic.

H atoms bonded to N atoms were located in a difference map and refined with distance restraints of N7—H7 and N13—H13 (molecule 1), N7A—H7A and N13A—H13A (molecule 2) = 0.88 (2) Å, and with *U*_iso_(H) = 1.2*U*_eq_(N). In the water molecule, H atoms were also located in a difference map and refined with distance restraints of O1—H1A and O1—H1B = 0.84 (2) Å, and with *U*_iso_(H) = 1.5*U*_eq_(O). Other H atoms were positioned geometrically and refined using a riding model with C—H = 0.95–0.99 Å and with *U*_iso_(H) = 1.2 *U*_eq_(C) or 1.5 *U*_eq_(methyl C).

### Crystal data

**Table:**

2C_14_H_22_N_4_O_2_·0.5C_6_H_14_·H_2_O	*Z* = 2
*M**_r_* = 617.81	*F*(000) = 670
Triclinic, *P*1	*D*_x_ = 1.150 Mg m^−^^3^
*a* = 10.6055 (5) Å	Mo *K*α radiation, λ = 0.71073 Å
*b* = 12.2181 (6) Å	Cell parameters from 7150 reflections
*c* = 14.9774 (7) Å	θ = 0.4–28.3°
α = 88.060 (3)°	µ = 0.08 mm^−^^1^
β = 73.093 (4)°	*T* = 123 K
γ = 74.179 (3)°	Needle, colourless
*V* = 1784.36 (16) Å^3^	0.30 × 0.05 × 0.04 mm

### Data collection

**Table:**

Bruker–Nonius KappaCCD with APEXII detector diffractometer	6422 independent reflections
Radiation source: fine-focus sealed tube	3597 reflections with *I* > 2σ(*I*)
Graphite monochromator	*R*_int_ = 0.100
Detector resolution: 9 pixels mm^-1^	θ_max_ = 25.3°, θ_min_ = 2.3°
φ and ω scans	*h* = −12→12
Absorption correction: multi-scan (*SADABS*; Sheldrick, 2004)	*k* = −14→14
*T*_min_ = 0.977, *T*_max_ = 0.997	*l* = −16→17
21621 measured reflections	

### Refinement

**Table:**

Refinement on *F*^2^	Primary atom site location: structure-invariant direct methods
Least-squares matrix: full	Secondary atom site location: difference Fourier map
*R*[*F*^2^ > 2σ(*F*^2^)] = 0.079	Hydrogen site location: inferred from neighbouring sites
*wR*(*F*^2^) = 0.164	H atoms treated by a mixture of independent and constrained refinement
*S* = 1.04	*w* = 1/[σ^2^(*F*_o_^2^) + (0.0128*P*)^2^ + 3.1862*P*] where *P* = (*F*_o_^2^ + 2*F*_c_^2^)/3
6422 reflections	(Δ/σ)_max_ = 0.001
451 parameters	Δρ_max_ = 0.35 e Å^−^^3^
101 restraints	Δρ_min_ = −0.24 e Å^−^^3^

### Special details

**Table:**

Geometry. All e.s.d.'s (except the e.s.d. in the dihedral angle between two l.s. planes) are estimated using the full covariance matrix. The cell e.s.d.'s are taken into account individually in the estimation of e.s.d.'s in distances, angles and torsion angles; correlations between e.s.d.'s in cell parameters are only used when they are defined by crystal symmetry. An approximate (isotropic) treatment of cell e.s.d.'s is used for estimating e.s.d.'s involving l.s. planes.

### Fractional atomic coordinates and isotropic or equivalent isotropic displacement parameters (Å^2^)

**Table:**

	*x*	*y*	*z*	*U*_iso_*/*U*_eq_	Occ. (<1)
O8	0.3412 (3)	0.46261 (19)	0.05984 (17)	0.0307 (6)	
O14	0.4845 (3)	0.3749 (2)	0.46471 (17)	0.0358 (7)	
N1	0.3512 (3)	0.5088 (2)	0.2372 (2)	0.0233 (7)	
N3	0.3818 (3)	0.6952 (2)	0.2389 (2)	0.0285 (8)	
N7	0.3042 (3)	0.6391 (2)	0.1249 (2)	0.0256 (7)	
H7	0.287 (4)	0.7128 (17)	0.117 (2)	0.031*	
N13	0.4056 (3)	0.3746 (2)	0.3391 (2)	0.0262 (7)	
H13	0.395 (4)	0.333 (3)	0.297 (2)	0.031*	
C2	0.3483 (4)	0.6108 (3)	0.2042 (2)	0.0234 (8)	
C4	0.4134 (4)	0.6713 (3)	0.3196 (3)	0.0304 (9)	
H4	0.4324	0.7296	0.3496	0.037*	
C5	0.4204 (4)	0.5693 (3)	0.3616 (3)	0.0266 (9)	
H5	0.4425	0.5559	0.4188	0.032*	
C6	0.3925 (4)	0.4870 (3)	0.3142 (2)	0.0240 (8)	
C8	0.3040 (4)	0.5661 (3)	0.0571 (2)	0.0235 (8)	
C9	0.2520 (4)	0.6243 (3)	−0.0223 (2)	0.0241 (8)	
C10	0.3278 (5)	0.7128 (4)	−0.0646 (3)	0.0516 (13)	
H10A	0.3121	0.7713	−0.0163	0.077*	
H10B	0.2935	0.7483	−0.1157	0.077*	
H10C	0.4262	0.6754	−0.0887	0.077*	
C11	0.0990 (4)	0.6824 (4)	0.0157 (3)	0.0479 (12)	
H11A	0.0835	0.7404	0.0642	0.072*	
H11B	0.0506	0.6256	0.0424	0.072*	
H11C	0.0644	0.7184	−0.0352	0.072*	
C12	0.2773 (5)	0.5344 (3)	−0.0978 (3)	0.0490 (12)	
H12A	0.2445	0.5706	−0.1493	0.073*	
H12B	0.2281	0.4780	−0.0717	0.073*	
H12C	0.3757	0.4966	−0.1211	0.073*	
C14	0.4569 (4)	0.3226 (3)	0.4087 (2)	0.0249 (8)	
C15	0.4773 (4)	0.1939 (3)	0.4109 (3)	0.0348 (10)	
C16	0.3527 (12)	0.1542 (14)	0.4115 (16)	0.049 (4)	0.6
H16A	0.3759	0.0709	0.4125	0.074*	0.6
H16B	0.3252	0.1788	0.3554	0.074*	0.6
H16C	0.2769	0.1873	0.4672	0.074*	0.6
C17	0.580 (3)	0.145 (2)	0.3155 (10)	0.051 (4)	0.6
H17A	0.5968	0.0616	0.3125	0.077*	0.6
H17B	0.6658	0.1637	0.3080	0.077*	0.6
H17C	0.5409	0.1770	0.2653	0.077*	0.6
C18	0.5496 (13)	0.1468 (16)	0.4841 (8)	0.055 (4)	0.6
H18A	0.5631	0.0642	0.4858	0.083*	0.6
H18B	0.4934	0.1826	0.5455	0.083*	0.6
H18C	0.6388	0.1632	0.4680	0.083*	0.6
C16B	0.3332 (17)	0.182 (2)	0.425 (2)	0.049 (5)	0.4
H16G	0.3356	0.1015	0.4267	0.074*	0.4
H16H	0.2996	0.2159	0.3728	0.074*	0.4
H16I	0.2719	0.2221	0.4837	0.074*	0.4
C17B	0.596 (4)	0.128 (3)	0.3298 (18)	0.057 (6)	0.4
H17G	0.6042	0.0467	0.3344	0.085*	0.4
H17H	0.6810	0.1432	0.3322	0.085*	0.4
H17I	0.5782	0.1533	0.2706	0.085*	0.4
C18B	0.493 (2)	0.162 (2)	0.5081 (10)	0.057 (5)	0.4
H18G	0.5070	0.0797	0.5141	0.086*	0.4
H18H	0.4102	0.2028	0.5563	0.086*	0.4
H18I	0.5723	0.1829	0.5158	0.086*	0.4
O8A	0.5012 (3)	0.06266 (19)	0.11110 (18)	0.0308 (6)	
O14A	−0.1719 (3)	0.2917 (2)	0.2818 (2)	0.0401 (7)	
N1A	0.2274 (3)	0.0794 (2)	0.2012 (2)	0.0241 (7)	
N3A	0.1930 (3)	−0.0951 (2)	0.1596 (2)	0.0291 (8)	
N7A	0.4086 (3)	−0.0859 (2)	0.1563 (2)	0.0278 (7)	
H7A	0.424 (4)	−0.1590 (17)	0.163 (3)	0.033*	
N13A	0.0596 (3)	0.2457 (2)	0.2438 (2)	0.0278 (7)	
H13A	0.134 (3)	0.268 (3)	0.238 (3)	0.033*	
C2A	0.2704 (4)	−0.0302 (3)	0.1724 (2)	0.0246 (8)	
C4A	0.0594 (4)	−0.0408 (3)	0.1783 (3)	0.0319 (9)	
H4A	−0.0004	−0.0836	0.1721	0.038*	
C5A	0.0030 (4)	0.0726 (3)	0.2060 (3)	0.0296 (9)	
H5A	−0.0920	0.1087	0.2172	0.035*	
C6A	0.0941 (4)	0.1311 (3)	0.2166 (2)	0.0258 (9)	
C8A	0.5165 (4)	−0.0389 (3)	0.1257 (2)	0.0236 (8)	
C9A	0.6589 (4)	−0.1224 (3)	0.1082 (2)	0.0245 (8)	
C10A	0.6809 (4)	−0.2132 (3)	0.0330 (3)	0.0382 (10)	
H10D	0.7725	−0.2662	0.0219	0.057*	
H10E	0.6728	−0.1764	−0.0250	0.057*	
H10F	0.6116	−0.2550	0.0541	0.057*	
C11A	0.6756 (4)	−0.1802 (3)	0.1980 (3)	0.0378 (10)	
H11D	0.7674	−0.2333	0.1852	0.057*	
H11E	0.6065	−0.2219	0.2205	0.057*	
H11F	0.6638	−0.1223	0.2457	0.057*	
C12A	0.7650 (4)	−0.0561 (3)	0.0739 (3)	0.0340 (10)	
H12D	0.8572	−0.1083	0.0619	0.051*	
H12E	0.7513	0.0024	0.1217	0.051*	
H12F	0.7548	−0.0196	0.0162	0.051*	
C14A	−0.0697 (4)	0.3197 (3)	0.2778 (2)	0.0283 (9)	
C15A	−0.0712 (4)	0.4379 (3)	0.3093 (3)	0.0303 (9)	
C16A	0.0042 (5)	0.4278 (4)	0.3835 (3)	0.0526 (13)	
H16D	0.0024	0.5039	0.4034	0.079*	
H16E	0.0994	0.3825	0.3573	0.079*	
H16F	−0.0411	0.3904	0.4375	0.079*	
C17A	0.0004 (4)	0.4968 (3)	0.2252 (3)	0.0396 (11)	
H17D	−0.0007	0.5725	0.2455	0.059*	
H17E	−0.0480	0.5043	0.1776	0.059*	
H17F	0.0954	0.4511	0.1987	0.059*	
C18A	−0.2197 (4)	0.5074 (3)	0.3492 (3)	0.0453 (11)	
H18D	−0.2227	0.5835	0.3699	0.068*	
H18E	−0.2655	0.4693	0.4023	0.068*	
H18F	−0.2666	0.5141	0.3009	0.068*	
O1	0.3274 (3)	0.2837 (2)	0.19608 (18)	0.0267 (6)	
H1A	0.367 (4)	0.219 (2)	0.168 (2)	0.040*	
H1B	0.341 (4)	0.336 (3)	0.161 (2)	0.040*	
C19	0.0268 (6)	0.7745 (4)	0.3857 (4)	0.0705 (16)	
H19A	0.0798	0.6973	0.3935	0.106*	0.6
H19B	0.0354	0.7854	0.3193	0.106*	0.6
H19C	−0.0699	0.7851	0.4205	0.106*	0.6
H19D	0.0647	0.6924	0.3894	0.106*	0.4
H19E	0.0794	0.8003	0.3278	0.106*	0.4
H19F	−0.0692	0.7906	0.3865	0.106*	0.4
C20	0.0808 (9)	0.8604 (8)	0.4226 (8)	0.062 (3)	0.6
H20A	0.1341	0.8212	0.4644	0.074*	0.6
H20B	0.1455	0.8850	0.3690	0.074*	0.6
C20B	0.0356 (16)	0.8381 (9)	0.4706 (8)	0.057 (4)	0.4
H20C	−0.0158	0.8098	0.5287	0.068*	0.4
H20D	0.1327	0.8194	0.4701	0.068*	0.4
C21	−0.0176 (6)	0.9607 (4)	0.4725 (4)	0.0702 (16)	
H21A	−0.0932	0.9351	0.5157	0.084*	0.6
H21B	−0.0562	1.0066	0.4261	0.084*	0.6
H21C	−0.1187	0.9775	0.4932	0.084*	0.4
H21D	0.0081	0.9829	0.4069	0.084*	0.4

### Atomic displacement parameters (Å^2^)

**Table:**

	*U*^11^	*U*^22^	*U*^33^	*U*^12^	*U*^13^	*U*^23^
O8	0.0437 (18)	0.0156 (13)	0.0338 (15)	−0.0028 (12)	−0.0177 (13)	0.0011 (11)
O14	0.0485 (19)	0.0318 (15)	0.0313 (15)	−0.0069 (13)	−0.0216 (14)	−0.0006 (12)
N1	0.0280 (19)	0.0180 (15)	0.0295 (17)	−0.0078 (13)	−0.0159 (14)	0.0030 (13)
N3	0.034 (2)	0.0175 (16)	0.0382 (19)	−0.0080 (14)	−0.0167 (16)	0.0020 (14)
N7	0.032 (2)	0.0144 (15)	0.0355 (18)	−0.0059 (14)	−0.0184 (15)	0.0034 (14)
N13	0.039 (2)	0.0172 (16)	0.0276 (17)	−0.0072 (14)	−0.0189 (15)	0.0037 (13)
C2	0.027 (2)	0.0165 (18)	0.030 (2)	−0.0055 (16)	−0.0129 (17)	0.0023 (15)
C4	0.031 (2)	0.023 (2)	0.040 (2)	−0.0082 (17)	−0.0135 (19)	−0.0071 (17)
C5	0.029 (2)	0.025 (2)	0.030 (2)	−0.0089 (17)	−0.0129 (18)	0.0015 (16)
C6	0.023 (2)	0.0225 (19)	0.026 (2)	−0.0062 (16)	−0.0078 (17)	0.0005 (16)
C8	0.019 (2)	0.023 (2)	0.027 (2)	−0.0051 (16)	−0.0056 (16)	0.0030 (16)
C9	0.026 (2)	0.0224 (18)	0.0233 (19)	−0.0047 (16)	−0.0092 (16)	0.0057 (15)
C10	0.068 (3)	0.045 (3)	0.053 (3)	−0.028 (2)	−0.026 (3)	0.023 (2)
C11	0.034 (3)	0.061 (3)	0.042 (3)	0.004 (2)	−0.016 (2)	0.010 (2)
C12	0.068 (3)	0.036 (2)	0.039 (2)	0.004 (2)	−0.027 (2)	−0.002 (2)
C14	0.021 (2)	0.0254 (19)	0.026 (2)	−0.0057 (16)	−0.0052 (17)	0.0038 (16)
C15	0.046 (3)	0.025 (2)	0.046 (2)	−0.0164 (18)	−0.027 (2)	0.0111 (18)
C16	0.047 (6)	0.034 (8)	0.084 (8)	−0.021 (5)	−0.036 (6)	0.010 (6)
C17	0.055 (7)	0.016 (6)	0.081 (7)	0.006 (4)	−0.033 (5)	0.000 (5)
C18	0.069 (8)	0.041 (7)	0.082 (7)	−0.025 (7)	−0.056 (6)	0.032 (6)
C16B	0.056 (8)	0.026 (9)	0.063 (9)	−0.024 (7)	−0.003 (7)	0.017 (7)
C17B	0.050 (10)	0.031 (11)	0.088 (11)	−0.007 (8)	−0.020 (10)	0.002 (9)
C18B	0.087 (13)	0.044 (8)	0.058 (8)	−0.015 (11)	−0.052 (8)	0.020 (7)
O8A	0.0267 (16)	0.0167 (13)	0.0484 (16)	−0.0057 (11)	−0.0105 (13)	0.0062 (11)
O14A	0.0256 (17)	0.0328 (16)	0.061 (2)	−0.0081 (13)	−0.0100 (14)	−0.0021 (13)
N1A	0.0225 (19)	0.0166 (15)	0.0345 (17)	−0.0053 (13)	−0.0103 (14)	0.0016 (13)
N3A	0.030 (2)	0.0208 (16)	0.0425 (19)	−0.0107 (15)	−0.0159 (16)	0.0030 (14)
N7A	0.0260 (19)	0.0146 (15)	0.047 (2)	−0.0060 (14)	−0.0175 (16)	0.0045 (14)
N13A	0.023 (2)	0.0195 (16)	0.0410 (19)	−0.0089 (14)	−0.0061 (16)	−0.0016 (14)
C2A	0.026 (2)	0.0202 (19)	0.030 (2)	−0.0070 (17)	−0.0110 (17)	0.0066 (16)
C4A	0.031 (3)	0.026 (2)	0.047 (2)	−0.0133 (18)	−0.019 (2)	0.0019 (18)
C5A	0.022 (2)	0.025 (2)	0.043 (2)	−0.0055 (17)	−0.0113 (18)	−0.0041 (17)
C6A	0.026 (2)	0.0202 (19)	0.031 (2)	−0.0051 (17)	−0.0103 (17)	0.0018 (16)
C8A	0.026 (2)	0.0194 (19)	0.0271 (19)	−0.0041 (16)	−0.0131 (17)	0.0006 (15)
C9A	0.024 (2)	0.0181 (18)	0.033 (2)	−0.0031 (15)	−0.0123 (17)	0.0007 (15)
C10A	0.039 (3)	0.025 (2)	0.049 (3)	0.0015 (18)	−0.019 (2)	−0.0084 (18)
C11A	0.038 (3)	0.033 (2)	0.043 (2)	−0.0048 (19)	−0.018 (2)	0.0049 (19)
C12A	0.024 (2)	0.033 (2)	0.043 (2)	−0.0019 (18)	−0.0111 (19)	−0.0002 (18)
C14A	0.025 (2)	0.029 (2)	0.027 (2)	−0.0035 (18)	−0.0050 (17)	0.0010 (16)
C15A	0.028 (2)	0.029 (2)	0.033 (2)	−0.0038 (17)	−0.0112 (18)	−0.0036 (17)
C16A	0.068 (4)	0.043 (3)	0.051 (3)	−0.007 (2)	−0.030 (3)	−0.008 (2)
C17A	0.039 (3)	0.024 (2)	0.052 (3)	−0.0083 (19)	−0.008 (2)	−0.0008 (19)
C18A	0.036 (3)	0.034 (2)	0.053 (3)	−0.003 (2)	0.002 (2)	−0.013 (2)
O1	0.0328 (17)	0.0163 (13)	0.0333 (15)	−0.0067 (12)	−0.0131 (13)	0.0009 (11)
C19	0.073 (4)	0.056 (3)	0.070 (4)	−0.025 (3)	0.005 (3)	−0.013 (3)
C20	0.038 (6)	0.059 (6)	0.075 (7)	−0.017 (5)	0.007 (5)	−0.020 (6)
C20B	0.049 (10)	0.041 (7)	0.062 (9)	−0.014 (6)	0.010 (7)	0.008 (7)
C21	0.075 (4)	0.059 (3)	0.077 (4)	−0.021 (3)	−0.019 (3)	−0.007 (3)

### Geometric parameters (Å, º)

**Table:**

O8—C8	1.221 (4)	N3A—C4A	1.340 (5)
O14—C14	1.216 (4)	N7A—C8A	1.373 (5)
N1—C2	1.321 (4)	N7A—C2A	1.390 (5)
N1—C6	1.343 (4)	N7A—H7A	0.871 (18)
N3—C2	1.343 (4)	N13A—C14A	1.381 (5)
N3—C4	1.347 (5)	N13A—C6A	1.391 (4)
N7—C8	1.375 (4)	N13A—H13A	0.886 (18)
N7—C2	1.398 (5)	C4A—C5A	1.375 (5)
N7—H7	0.881 (18)	C4A—H4A	0.9500
N13—C14	1.372 (5)	C5A—C6A	1.394 (5)
N13—C6	1.392 (4)	C5A—H5A	0.9500
N13—H13	0.870 (18)	C8A—C9A	1.529 (5)
C4—C5	1.370 (5)	C9A—C12A	1.523 (5)
C4—H4	0.9500	C9A—C10A	1.530 (5)
C5—C6	1.391 (5)	C9A—C11A	1.531 (5)
C5—H5	0.9500	C10A—H10D	0.9800
C8—C9	1.528 (5)	C10A—H10E	0.9800
C9—C12	1.517 (5)	C10A—H10F	0.9800
C9—C11	1.524 (5)	C11A—H11D	0.9800
C9—C10	1.532 (5)	C11A—H11E	0.9800
C10—H10A	0.9800	C11A—H11F	0.9800
C10—H10B	0.9800	C12A—H12D	0.9800
C10—H10C	0.9800	C12A—H12E	0.9800
C11—H11A	0.9800	C12A—H12F	0.9800
C11—H11B	0.9800	C14A—C15A	1.529 (5)
C11—H11C	0.9800	C15A—C18A	1.522 (5)
C12—H12A	0.9800	C15A—C16A	1.531 (5)
C12—H12B	0.9800	C15A—C17A	1.537 (5)
C12—H12C	0.9800	C16A—H16D	0.9800
C14—C15	1.529 (5)	C16A—H16E	0.9800
C15—C16	1.524 (10)	C16A—H16F	0.9800
C15—C17B	1.525 (13)	C17A—H17D	0.9800
C15—C18	1.526 (10)	C17A—H17E	0.9800
C15—C16B	1.527 (13)	C17A—H17F	0.9800
C15—C18B	1.539 (12)	C18A—H18D	0.9800
C15—C17	1.543 (10)	C18A—H18E	0.9800
C16—H16A	0.9800	C18A—H18F	0.9800
C16—H16B	0.9800	O1—H1A	0.841 (19)
C16—H16C	0.9800	O1—H1B	0.828 (18)
C17—H17A	0.9800	C19—C20	1.512 (9)
C17—H17B	0.9800	C19—C20B	1.550 (12)
C17—H17C	0.9800	C19—H19A	0.9800
C18—H18A	0.9800	C19—H19B	0.9800
C18—H18B	0.9800	C19—H19C	0.9800
C18—H18C	0.9800	C19—H19D	0.9800
C16B—H16G	0.9800	C19—H19E	0.9800
C16B—H16H	0.9800	C19—H19F	0.9800
C16B—H16I	0.9800	C20—C21	1.435 (9)
C17B—H17G	0.9800	C20—H20A	0.9900
C17B—H17H	0.9800	C20—H20B	0.9900
C17B—H17I	0.9800	C20B—C21	1.447 (11)
C18B—H18G	0.9800	C20B—H20C	0.9900
C18B—H18H	0.9800	C20B—H20D	0.9900
C18B—H18I	0.9800	C21—C21^i^	1.473 (10)
O8A—C8A	1.227 (4)	C21—H21A	0.9900
O14A—C14A	1.208 (4)	C21—H21B	0.9900
N1A—C2A	1.334 (4)	C21—H21C	0.9900
N1A—C6A	1.336 (5)	C21—H21D	0.9900
N3A—C2A	1.339 (4)		
			
C2—N1—C6	116.2 (3)	C6A—N13A—H13A	111 (3)
C2—N3—C4	113.6 (3)	N1A—C2A—N3A	126.7 (3)
C8—N7—C2	127.7 (3)	N1A—C2A—N7A	117.9 (3)
C8—N7—H7	120 (2)	N3A—C2A—N7A	115.3 (3)
C2—N7—H7	112 (2)	N3A—C4A—C5A	124.4 (3)
C14—N13—C6	127.1 (3)	N3A—C4A—H4A	117.8
C14—N13—H13	120 (2)	C5A—C4A—H4A	117.8
C6—N13—H13	112 (2)	C4A—C5A—C6A	115.8 (4)
N1—C2—N3	127.5 (3)	C4A—C5A—H5A	122.1
N1—C2—N7	118.9 (3)	C6A—C5A—H5A	122.1
N3—C2—N7	113.7 (3)	N1A—C6A—N13A	112.8 (3)
N3—C4—C5	125.0 (3)	N1A—C6A—C5A	121.7 (3)
N3—C4—H4	117.5	N13A—C6A—C5A	125.5 (3)
C5—C4—H4	117.5	O8A—C8A—N7A	122.8 (3)
C4—C5—C6	114.9 (3)	O8A—C8A—C9A	121.7 (3)
C4—C5—H5	122.5	N7A—C8A—C9A	115.4 (3)
C6—C5—H5	122.5	C12A—C9A—C8A	108.2 (3)
N1—C6—C5	122.4 (3)	C12A—C9A—C10A	109.1 (3)
N1—C6—N13	112.6 (3)	C8A—C9A—C10A	109.9 (3)
C5—C6—N13	124.9 (3)	C12A—C9A—C11A	109.5 (3)
O8—C8—N7	123.0 (3)	C8A—C9A—C11A	110.8 (3)
O8—C8—C9	122.1 (3)	C10A—C9A—C11A	109.4 (3)
N7—C8—C9	114.9 (3)	C9A—C10A—H10D	109.5
C12—C9—C11	110.0 (3)	C9A—C10A—H10E	109.5
C12—C9—C8	108.6 (3)	H10D—C10A—H10E	109.5
C11—C9—C8	108.9 (3)	C9A—C10A—H10F	109.5
C12—C9—C10	108.8 (3)	H10D—C10A—H10F	109.5
C11—C9—C10	109.6 (3)	H10E—C10A—H10F	109.5
C8—C9—C10	110.9 (3)	C9A—C11A—H11D	109.5
C9—C10—H10A	109.5	C9A—C11A—H11E	109.5
C9—C10—H10B	109.5	H11D—C11A—H11E	109.5
H10A—C10—H10B	109.5	C9A—C11A—H11F	109.5
C9—C10—H10C	109.5	H11D—C11A—H11F	109.5
H10A—C10—H10C	109.5	H11E—C11A—H11F	109.5
H10B—C10—H10C	109.5	C9A—C12A—H12D	109.5
C9—C11—H11A	109.5	C9A—C12A—H12E	109.5
C9—C11—H11B	109.5	H12D—C12A—H12E	109.5
H11A—C11—H11B	109.5	C9A—C12A—H12F	109.5
C9—C11—H11C	109.5	H12D—C12A—H12F	109.5
H11A—C11—H11C	109.5	H12E—C12A—H12F	109.5
H11B—C11—H11C	109.5	O14A—C14A—N13A	121.9 (3)
C9—C12—H12A	109.5	O14A—C14A—C15A	123.7 (3)
C9—C12—H12B	109.5	N13A—C14A—C15A	114.4 (3)
H12A—C12—H12B	109.5	C18A—C15A—C14A	108.4 (3)
C9—C12—H12C	109.5	C18A—C15A—C16A	110.2 (3)
H12A—C12—H12C	109.5	C14A—C15A—C16A	110.1 (3)
H12B—C12—H12C	109.5	C18A—C15A—C17A	109.6 (3)
O14—C14—N13	122.6 (3)	C14A—C15A—C17A	109.6 (3)
O14—C14—C15	122.2 (3)	C16A—C15A—C17A	109.0 (4)
N13—C14—C15	115.2 (3)	C15A—C16A—H16D	109.5
C16—C15—C18	117.0 (11)	C15A—C16A—H16E	109.5
C17B—C15—C16B	120 (2)	H16D—C16A—H16E	109.5
C16—C15—C14	115.5 (7)	C15A—C16A—H16F	109.5
C17B—C15—C14	112.8 (18)	H16D—C16A—H16F	109.5
C18—C15—C14	109.1 (8)	H16E—C16A—H16F	109.5
C16B—C15—C14	103.2 (10)	C15A—C17A—H17D	109.5
C17B—C15—C18B	114.3 (16)	C15A—C17A—H17E	109.5
C16B—C15—C18B	99.5 (15)	H17D—C17A—H17E	109.5
C14—C15—C18B	105.2 (12)	C15A—C17A—H17F	109.5
C16—C15—C17	102.4 (16)	H17D—C17A—H17F	109.5
C18—C15—C17	106.1 (11)	H17E—C17A—H17F	109.5
C14—C15—C17	105.5 (11)	C15A—C18A—H18D	109.5
C15—C16—H16A	109.5	C15A—C18A—H18E	109.5
C15—C16—H16B	109.5	H18D—C18A—H18E	109.5
H16A—C16—H16B	109.5	C15A—C18A—H18F	109.5
C15—C16—H16C	109.5	H18D—C18A—H18F	109.5
H16A—C16—H16C	109.5	H18E—C18A—H18F	109.5
H16B—C16—H16C	109.5	H1A—O1—H1B	111 (4)
C15—C17—H17A	109.5	C20—C19—H19A	109.5
C15—C17—H17B	109.5	C20—C19—H19B	109.5
H17A—C17—H17B	109.5	H19A—C19—H19B	109.5
C15—C17—H17C	109.5	C20—C19—H19C	109.5
H17A—C17—H17C	109.5	H19A—C19—H19C	109.5
H17B—C17—H17C	109.5	H19B—C19—H19C	109.5
C15—C18—H18A	109.5	C20B—C19—H19D	109.5
C15—C18—H18B	109.5	C20B—C19—H19E	109.5
H18A—C18—H18B	109.5	H19D—C19—H19E	109.5
C15—C18—H18C	109.5	C20B—C19—H19F	109.5
H18A—C18—H18C	109.5	H19D—C19—H19F	109.5
H18B—C18—H18C	109.5	H19E—C19—H19F	109.5
C15—C16B—H16G	109.5	C21—C20—C19	117.4 (7)
C15—C16B—H16H	109.5	C21—C20—H20A	107.9
H16G—C16B—H16H	109.5	C19—C20—H20A	107.9
C15—C16B—H16I	109.5	C21—C20—H20B	107.9
H16G—C16B—H16I	109.5	C19—C20—H20B	107.9
H16H—C16B—H16I	109.5	H20A—C20—H20B	107.2
C15—C17B—H17G	109.5	C21—C20B—C19	114.3 (9)
C15—C17B—H17H	109.5	C21—C20B—H20C	108.7
H17G—C17B—H17H	109.5	C19—C20B—H20C	108.7
C15—C17B—H17I	109.5	C21—C20B—H20D	108.7
H17G—C17B—H17I	109.5	C19—C20B—H20D	108.7
H17H—C17B—H17I	109.5	H20C—C20B—H20D	107.6
C15—C18B—H18G	109.5	C20—C21—C21^i^	122.8 (7)
C15—C18B—H18H	109.5	C20B—C21—C21^i^	123.2 (8)
H18G—C18B—H18H	109.5	C20—C21—H21A	106.6
C15—C18B—H18I	109.5	C21^i^—C21—H21A	106.6
H18G—C18B—H18I	109.5	C20—C21—H21B	106.6
H18H—C18B—H18I	109.5	C21^i^—C21—H21B	106.6
C2A—N1A—C6A	116.9 (3)	H21A—C21—H21B	106.6
C2A—N3A—C4A	114.4 (3)	C20B—C21—H21C	106.5
C8A—N7A—C2A	126.9 (3)	C21^i^—C21—H21C	106.5
C8A—N7A—H7A	120 (3)	C20B—C21—H21D	106.5
C2A—N7A—H7A	113 (3)	C21^i^—C21—H21D	106.5
C14A—N13A—C6A	127.9 (3)	H21C—C21—H21D	106.5
C14A—N13A—H13A	121 (2)		
			
C6—N1—C2—N3	−0.1 (6)	C6A—N1A—C2A—N3A	−1.8 (5)
C6—N1—C2—N7	−179.1 (3)	C6A—N1A—C2A—N7A	−179.7 (3)
C4—N3—C2—N1	−4.2 (5)	C4A—N3A—C2A—N1A	0.0 (5)
C4—N3—C2—N7	174.9 (3)	C4A—N3A—C2A—N7A	177.9 (3)
C8—N7—C2—N1	−23.1 (6)	C8A—N7A—C2A—N1A	−31.5 (5)
C8—N7—C2—N3	157.8 (3)	C8A—N7A—C2A—N3A	150.3 (3)
C2—N3—C4—C5	3.9 (6)	C2A—N3A—C4A—C5A	1.8 (5)
N3—C4—C5—C6	0.4 (6)	N3A—C4A—C5A—C6A	−1.7 (6)
C2—N1—C6—C5	5.0 (5)	C2A—N1A—C6A—N13A	−178.2 (3)
C2—N1—C6—N13	−174.1 (3)	C2A—N1A—C6A—C5A	1.9 (5)
C4—C5—C6—N1	−5.1 (5)	C14A—N13A—C6A—N1A	−169.9 (3)
C4—C5—C6—N13	173.9 (4)	C14A—N13A—C6A—C5A	10.0 (6)
C14—N13—C6—N1	173.8 (3)	C4A—C5A—C6A—N1A	−0.3 (5)
C14—N13—C6—C5	−5.3 (6)	C4A—C5A—C6A—N13A	179.9 (3)
C2—N7—C8—O8	1.9 (6)	C2A—N7A—C8A—O8A	2.1 (6)
C2—N7—C8—C9	−178.6 (3)	C2A—N7A—C8A—C9A	−176.3 (3)
O8—C8—C9—C12	−8.7 (5)	O8A—C8A—C9A—C12A	1.1 (5)
N7—C8—C9—C12	171.8 (3)	N7A—C8A—C9A—C12A	179.4 (3)
O8—C8—C9—C11	111.1 (4)	O8A—C8A—C9A—C10A	−117.9 (4)
N7—C8—C9—C11	−68.4 (4)	N7A—C8A—C9A—C10A	60.4 (4)
O8—C8—C9—C10	−128.2 (4)	O8A—C8A—C9A—C11A	121.0 (4)
N7—C8—C9—C10	52.3 (4)	N7A—C8A—C9A—C11A	−60.6 (4)
C6—N13—C14—O14	8.2 (6)	C6A—N13A—C14A—O14A	−5.3 (6)
C6—N13—C14—C15	−171.8 (3)	C6A—N13A—C14A—C15A	174.7 (3)
O14—C14—C15—C16	127.7 (10)	O14A—C14A—C15A—C18A	2.7 (5)
N13—C14—C15—C16	−52.3 (10)	N13A—C14A—C15A—C18A	−177.3 (3)
O14—C14—C15—C17B	−109 (2)	O14A—C14A—C15A—C16A	123.3 (4)
N13—C14—C15—C17B	71 (2)	N13A—C14A—C15A—C16A	−56.7 (4)
O14—C14—C15—C18	−6.4 (7)	O14A—C14A—C15A—C17A	−116.8 (4)
N13—C14—C15—C18	173.6 (6)	N13A—C14A—C15A—C17A	63.2 (4)
O14—C14—C15—C16B	120.1 (13)	C20B—C19—C20—C21	−64.2 (11)
N13—C14—C15—C16B	−59.9 (13)	C20—C19—C20B—C21	60.4 (10)
O14—C14—C15—C18B	16.2 (10)	C19—C20—C21—C20B	65.7 (12)
N13—C14—C15—C18B	−163.8 (9)	C19—C20—C21—C21^i^	167.6 (7)
O14—C14—C15—C17	−120.0 (13)	C19—C20B—C21—C20	−60.0 (10)
N13—C14—C15—C17	60.0 (13)	C19—C20B—C21—C21^i^	−160.5 (8)

Symmetry code: (i) −*x*, −*y*+2, −*z*+1.

### Hydrogen-bond geometry (Å, º)

**Table:**

*D*—H···*A*	*D*—H	H···*A*	*D*···*A*	*D*—H···*A*
O1—H1*A*···O8*A*	0.84 (2)	2.08 (2)	2.910 (3)	167 (4)
O1—H1*A*···N1*A*	0.84 (2)	2.51 (4)	2.958 (4)	115 (3)
O1—H1*B*···O8	0.83 (2)	2.13 (2)	2.943 (3)	168 (4)
O1—H1*B*···N1	0.83 (2)	2.48 (4)	2.931 (4)	115 (3)
N7—H7···N3*A*^ii^	0.88 (2)	2.32 (2)	3.144 (4)	156 (3)
N13—H13···O1	0.87 (2)	2.02 (2)	2.864 (4)	162 (4)
N7*A*—H7*A*···N3^iii^	0.87 (2)	2.16 (2)	2.958 (4)	152 (3)
N13*A*—H13*A*···O1	0.89 (2)	2.02 (2)	2.882 (4)	164 (4)
C5—H5···O14^iv^	0.95	2.37	3.205 (5)	147

Symmetry codes: (ii) *x*, *y*+1, *z*; (iii) *x*, *y*−1, *z*; (iv) −*x*+1, −*y*+1, −*z*+1.
